# Fucoidan and Lung Function: Value in Viral Infection

**DOI:** 10.3390/md19010004

**Published:** 2020-12-24

**Authors:** J. Helen Fitton, Ah Young Park, Samuel S. Karpiniec, Damien N. Stringer

**Affiliations:** Marinova Pty Ltd., 249 Kennedy Drive, Cambridge, TAS 7170, Australia; ahyoung.park@marinova.com.au (A.Y.P.); sam.karpiniec@marinova.com.au (S.S.K.); damien.stringer@marinova.com.au (D.N.S.)

**Keywords:** fucoidan, lung, fibrosis, respiratory, influenza, corona, COPD

## Abstract

Compromised lung function is a feature of both infection driven and non-infective pathologies. Viral infections—including the current pandemic strain SARS-CoV-2—that affect lung function can cause both acute and long-term chronic damage. SARS-CoV-2 infection suppresses innate immunity and promotes an inflammatory response. Targeting these aspects of SARS-CoV-2 is important as the pandemic affects greater proportions of the population. In clinical and animal studies, fucoidans have been shown to increase innate immunity and decrease inflammation. In addition, dietary fucoidan has been shown to attenuate pulmonary damage in a model of acute viral infection. Direct inhibition of SARS-CoV-2 in vitro has been described, but is not universal. This short review summarizes the current research on fucoidan with regard to viral lung infections and lung damage.

## 1. Introduction

Lung function is critical to life. Intake of air results in gas exchange at the delicate sacs in the lung called alveoli. The blood vessels that pass through the lungs carry deoxygenated blood. Oxygen is replenished and carbon dioxide is expelled. The lining of the lungs, lung epithelial cells, and the smooth muscle cells that help sacs expand and contract, are pivotal to these gas exchange functions [1]. Chronic lung damage caused by respiratory viral infections and ensuing bacterial infections may take a long time to heal, and sometimes leave permanent scarring. Once compromised it is difficult to rebuild damaged lung architecture.

The recent surge of interest in respiratory viral effects on lung function is a result of the pandemic coronavirus, SARS-CoV-2 as well as prior coronaviruses SARS-CoV and MERS-CoV [2,3]. Ventilators are a limited resource in hospitals. If lung function can be preserved sufficiently to limit the need for hospital treatment or ventilation, there is considerable benefit to both patient and society. A reduction in chronic post-infection lung damage would be valuable. A useful agent would support immune clearance of the virus, and limit inflammatory damage. Although interim evidence from the WHO solidarity therapeutics trial suggested that direct anti-viral agents do not reduce morbidity [4], agents that potentially inhibit viral entry and viral replication also have utility. Recent survivors of COVID 19 infection report ongoing health issues for months after the clearance of infection, or ‘long COVID’. SARS-CoV-2 inhibits innate immune responses and promotes strong inflammatory expression [5]. A recent review by Zabetakis et al. points out the inflammation link with SARS-CoV-2 and the role of diet in addressing poor nutritional status and preexisting diseases such as diabetes, chronic lung disease, cardiovascular disease, and obesity [6].

Dietary fucoidan has been shown to increase innate immunity in various models [7]. In a clinical study, ingestion of fucoidan was shown to restore a marker of gut innate immunity (lysozyme) to normal levels in athletes [8]. As a known selectin blockade agent, fucoidan has been shown to act as an anti-inflammatory [9,10]. The pharmacokinetics of fucoidan have been studied, and low levels of systemic uptake and excretion confirmed [11,12]. Seaweed derived polysaccharides have been noted for their potential to prevent or ameliorate viral infections. Three recent reviews have queried the potential of seaweed polysaccharides in the context of the current pandemic [2,13,14]. In vitro and in vivo data has demonstrated efficacy against infection by influenza viruses. Inhibitory activity specifically against SARS-CoV-2 infection in vitro has been noted for linear fucans from a sea urchin, galactans from red seaweed *Botryocladia occidentalis*, and a highly purified fucoidan fraction from *Saccharina japonica* [15,16]. However, commercially available fucoidans from *Fucus vesiculosus* and *Undaria pinnatifida* were not active in vitro [17]. In addition, computational screening of marine compounds from natural sources has indicated that polyphenolics (commonly co-extracted with fucoidan) from seaweeds may have inhibitory activity [18] although this has not been confirmed with experimental studies.

Research work on lung damage attenuation and antiviral activity is continuing. In this mini-review, we aim to provide a snapshot of current fucoidan research with respect to lung function.

## 2. Viral Infection of the Lung

Influenza and Corona virus strains can develop resistance to conventional drugs, meaning that there is need for broad spectrum strategies. Seaweed metabolites have recently been reviewed for potential in influenza infection [19]. Periera and Critchley have questioned the potential role of seaweed derived compounds as viral infection inhibitors in the current Corona virus pandemic, as has Itzhaki [2,13]. However, with conflicting current reports on direct anti-viral activity, a more relevant role specifically for fucoidan may be as either an immune modulating agent or a tissue sparing anti-inflammatory agent. As shown in Table 1, respiratory viral infections, including strains of influenza, have been shown to be inhibited in vitro and in vivo by orally delivered fucoidan [20,21]. Indirectly, oral fucoidan appears to have capacity to enhance innate immunity and increase responses to vaccines in the elderly. A modest 300 mg oral daily dose of fucoidan from *Undaria pinnatifida* enhanced vaccine responses [22].

A key feature of SARS-CoV-2 is a low initial innate immune response, allowing the viral infection to progress until a cytokine response becomes prevalent [5]. The high pro-inflammatory cues are a key contributor to COVID 19, and can progress to a cytokine storm. Even without the extremes of a cytokine storm, inflammatory damage that results from an immune response to a pathogen can cause permanent fibrotic changes to the lung. Oral fucoidan may be well placed as a dietary addition to reduce this inflammatory damage in the early stages. Richards et al. recently observed no viral reduction, but a significant reduction in gross lung pathology in a mouse model of severe influenza using orally delivered fucoidan [23]. Additional lung damage models are currently being investigated, to determine if this is connected to increased innate immune function, or/and a decreased inflammatory response.

Respiratory viruses including influenza, coronaviruses and RSV enter the respiratory system via receptor mediated mechanisms. Whilst some viruses attach to receptors in the upper tract, others tend to attach lower in the bronchi and the lung, depending on the cell surface receptors expressed [24]. Influenza viruses use hemagglutinin and neuraminidase to enter the cells, whereas Coronaviruses use S protein spikes attaching via the ACE2 receptor, with apparent coordination with additional heparin sulfate type receptors [25]. The differential expression of ACE2 receptors in children and smokers accounts in part for the differences in infection rates [26]. Intriguingly, SARS-CoV-2 also appears to intervene with the intrinsic immune response in the gut [27], curtailing the autocrine action of interferon, as was previously described by Blanco-Melo [5]. In this way, the virus curtails the immune responses in the gut and creates a reservoir for itself.

Recent research shows that fucoidan can restore a previously depressed lysozyme function in the gut [8], suggesting that it has the potential to attenuate this effect at the gut level. Additional data on orally delivered fucoidan indicates potential attenuating effects on viral lung damage described by Richards and Hayashi [20,23].

Fucoidans and other marine algal derived polysaccharides have shown direct inhibition of influenza virus entry to cells. This activity has been shown for fucoidan from different sources as shown in Table 1.

The recent pandemic with the Corona virus variant SARS-CoV-2 has been assessed in vitro with a variety of fucan, fucoidan, and heparin compounds. A key feature of COVID 19 infection is micro clotting, and the anticoagulant effects of heparin have proved lifesaving [29]. Heparin was additionally shown to directly inhibit SARS-CoV-2 in vitro, adding to its likely efficacy as a therapeutic agent [15,16]. The fucoidan fractions tested thus far are galactofucan laboratory samples, rather than commercially available preparations, but show interesting activity by preventing binding of viral spike protein to heparin but not to ACE2. Studies are outlined in Table 2.

By contrast, in recent screening of unfractionated fucoidan from *Fucus vesiculosus* and *Undaria pinnatifida,* no activity was noted in an in vitro infection model [17]. There are currently no clinical studies with fucoidan in this field.

## 3. Nonpathogenic Lung Diseases

Chronic obstructive pulmonary disease (COPD) is a collective name for irreversible lung damage caused by chronic diseases such as asthma and bronchitis, smoking, and air pollution. The fibrosis induced by COPD leaves scars in place of functional lung tissue.

Fucoidan may ameliorate a proportion of damage, acting as both an anti-inflammatory and inhibitor of epithelial-mesenchymal transformation. Fucoidan has been shown to protect against tobacco smoke MUC5 activation in bronchial cells [32] and inhibit Toll-like receptor mediated cytokine release in bronchial cells [33]. In unrelated cell types, fucoidan prevents epithelial mesenchymal transformation, preserving function in the tissues [34]. Bleomycin induced damage is attenuated by fucoidan in a mouse model [35]. The recent review by Chen et al. also discusses the potential roles for a range of plant polysaccharides in the attenuation of pulmonary fibrosis, with a particular focus on post viral damage [14]. Studies are outlined in Table 3. Clinical studies are needed in this area.

## 4. Conclusions

Respiratory viral infections can cause acute and chronic damage to the lung. The current pandemic corona virus SARS-CoV-2 inhibits innate immune responses and promotes strong inflammatory expression. Fucoidan preparations have potential as supplementary agents to limit damage subsequent to respiratory viral infections by restoring innate immune function and inhibiting inflammation. Additional studies are needed to explore this hypothesis. Direct in vitro inhibition of infection by fucoidan fractions in vitro is not universal. It remains essential to comply with public health information to prevent the spread of SARS-CoV-2 and other pathogens.

## Figures and Tables

**Table 1:** Fucoidan fractions and inhibition of influenza viruses in vitro and in vivo.

Fucoidan Type	Study Focus	Description	Dose	Reference
*Undaria pinnatifida* Unfractionated	H1N1 Deep lung influenza PR8	Lung damage limited by oral intake of fucoidan in treatment and prevention models. Not correlated to viral titre.	3.52 or 7.04 mg/day	[23]
*Undaria pinnatifida* Discrete fraction 9kDa	H1N1 (A/NWS/33)	Virus yield in the mucosa of immunocompetent and compromised mice was reduced and stimulated mucosal immunoresponse.	IC50- 15 µg/mL, 5 mg/day post infection	[20]
*Undaria pinnatifida* Discrete fraction 9 kDa	Avian influenza viruses (H5N3 and H7N2)	Suppressed virus yields and increased antibody production.	1 mg or 5 mg/day	[21]
*Kjellmaniella crassifolia* Unfractionated536 kDa	PR8 (H1D1), Cal09 (H1N1), Minnesota (H2N2) and TXD09 (H1N1)	Inhibits virus replication in vitro and has low risk of inducing drug resistance.	IC50 34 µg/mL 10 or 20 μg/day intranasal	[28]
*Undaria pinnatifida* Unfractionated	Response to vaccine containing A/Brisbane/59/2007, A/Uruguay/716/2007, B/Brisbane/60/2008	Clinical. Enhanced immune responses to seasonal influenza vaccine in the elderly.	300 mg/day 4 weeks pre and 19 weeks post inoculation	[22]

**Table 2:** Fucoidan and heparin activity against SARS-CoV-2.

Compounds	Study Focus	Description	Inhibition IC50	Reference
Fucoidan from *Fucus vesiculosus* and *Undaria pinnatifida* Unfractionated	Inhibition of SARS-CoV-2	Neither polysaccharide inhibits viral infection in vitro Vero 76 cells.	>100 µg/ml	[17]
Iota carrageenan(IC), chondroitin sulfate C(CS), sea cucumber polysaccharide (SCPS), fucoidan (species not stated)	Inhibition of SARS-CoV-2 in vitro	All three polysaccharides inhibit SARS-CoV-2 in vitro. SCPS can bind to S glycoprotein.	iC ≥ 125 μg/mL CS nil SCPS 9.10 μg/mL Fucoidan 15.6 μg/mL	[30]
Sulfated galactofucan from Saccharina japonica (1, 3-linked α-L-Fuc*p* residues sulfated at C4 and C2/C4 and 1, 3-linked α-L-Fuc*p* residues sulfated at C4 and branched with 1, 6-linked β-d-galacto-biose)	Binding study of SARS CoV-2spike glycoprotein (SGP) with heparin and ACE2	Sulfated galactofucan inhibited interaction between SARS-CoV-2 SGPs and heparin, but not ACE2.	IC 50 of 27 nM (for inhibition of interaction of SGP and heparin)	[31]
Heparin (~17 kDa), TriS-heparin (~18 kDa), LMW and HMW fucoidan from *Saccharina japonica* (structure, see above)	In vitro antiviral properties that target SARS-CoV-2 and binding of S-proteins of SARS-CoV-2 and docking study	Binding efficiency of the compounds correlated anti-viral activities. The most potent was the HMW fucoidan from *Saccharina japonica* in low nM.	HMWfucoidan 8.3 μg/mL LMWfucoidan16 μg/mL Heparin36 μg/mL Tris-heparin88 μg/mL	[16]
UFH, enoxaparin, 6-O-desulfated UFH, 6-O-desulfated enoxaparin, sulfated fucan from *Lytechnius variegatus* and sulfated galactan from *Botryocladia occidentalis*	Binding study of SGP, transduction efficiency of a third generation lentiviral (pLV) vector	pLV-S particles were neutralized with an IC_50_ of low ng to high µg/L.	UFH 599 pg/mL L.Var. 3.3 ng/mL B. Occ 5.4 ng/mL Desulf enoxaparin nil	[15]

**Table 3:** Fucoidan activity in nonpathogenic lung diseases.

Fucoidan Type	Study Focus	Description	Dose	Reference
*Undaria pinnatifida*	Particulate Matter (PM) induced allergic airway inflammation	Balb/c mice exposed to PM and reduced allergic asthma symptoms by attenuating the airway inflammatory response and mucus hypersecretion.	100, 400 mg/Kg/day	[36]
Probably *Fucus vesiculosis*	Effect on MUC5AC expression in a human bronchial epithelial cell line (NCI-H292)	In vitro fucoidan suppresses MUC5AC expression in human bronchial epithelial cells through deactivation of AP-1.	30 mg/mL fucoidan completely blocked induction of MUC5AC	[32,34]
*Ascophyllum nodosum*	Effects on human bronchial epithelial cells via Toll-like-receptor-3 (TLR3) induced cytokine release	In vitro fucoidan inhibits inflammatory mediators associated with TLR3.	1 mg/mL solution significantly inhibits cytokine release, PGE2	[32,33]
*Cladosiphon okamuranus*	Protection from high oxygen tension damage	In vivo Balb/c mice peroxia induced inflammation and morphological alterations were significantly attenuated in the mice treated with fucoidan. Atomization inhalation of fucoidan also reduced the hyperoxia induced expression of IL1, IL6 and TNFα, and the phosphorylation of ERK1/2.	100 μg/mL by atomization inhalation	[33,37]
*Fucus vesiculosus*	Inhibition of inflammatory enzymes COX 2, extends clotting times	HMW fucoidan inhibited COX-2 significantly with greater selectivity than synthetic drug indomethacin. It also inhibited hyaluronidase enzyme and dipeptidyl peptidase-IV(DPP-IV).	COX2: 4.3 _g mL Hyaluronidase:2.9 _g mL DPPIV: 1.11 _g mL	[3,37]
*Fucus vesiculosus*	LPS induced lung injury	In vivo LPS-induced acute lung injury in mice through regulating GSK-3beta-Nrf2 signaling pathway.	20, 40, or 80 mg/kg	[3,38]
